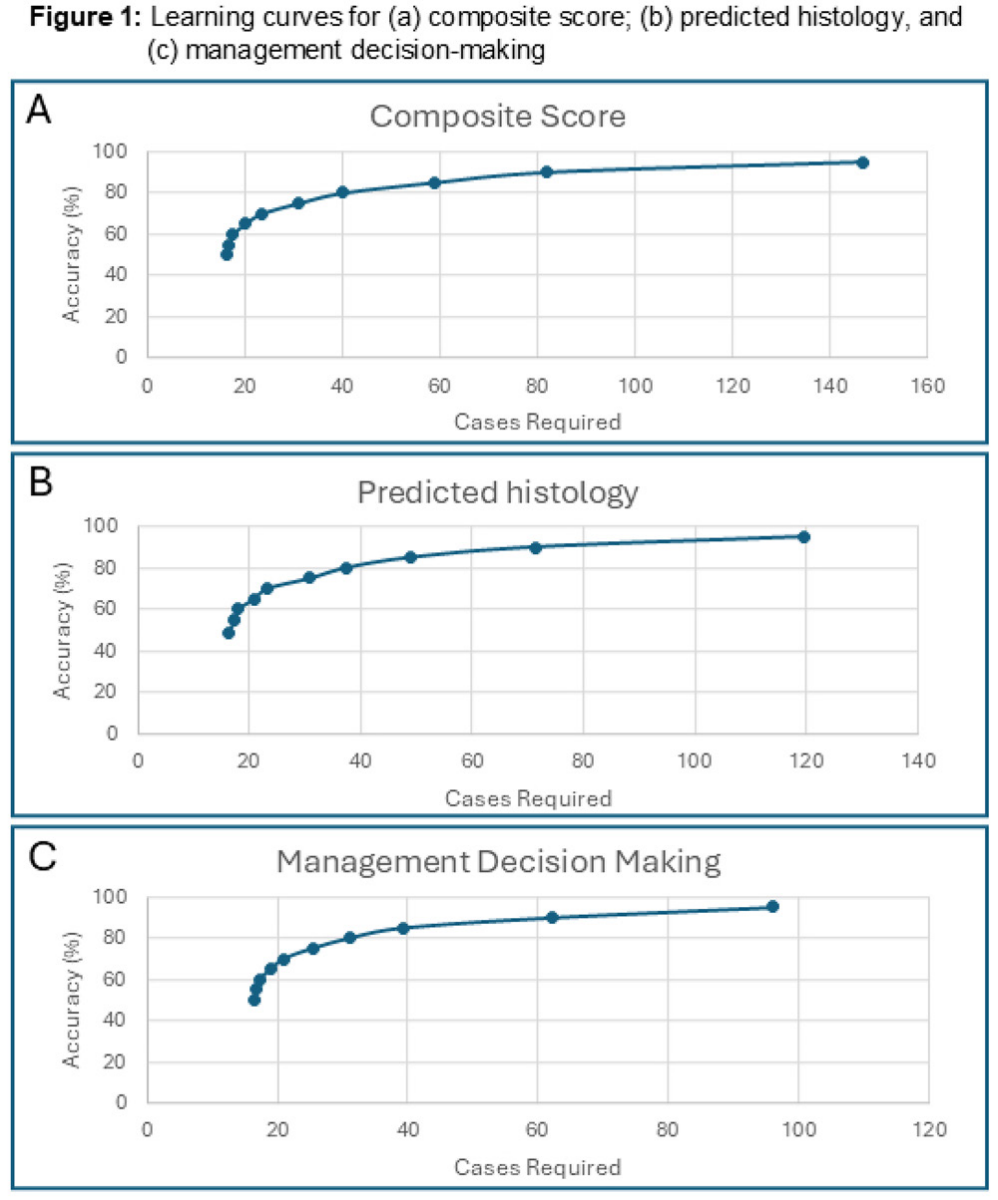# Poster Session I - A50 IMPROVING OPTICAL DIAGNOSIS AND MANAGEMENT DECISION-MAKING SKILLS THROUGH COGNITIVE SIMULATION WITH DELIBERATE PRACTICE: A PROSPECTIVE COHORT STUDY

**DOI:** 10.1093/jcag/gwaf042.050

**Published:** 2026-02-13

**Authors:** R Khan, K Boutis, R Bechara, S Grover, M Pusic, M Pecaric, C Menard, K Waschke, C M Walsh

**Affiliations:** University of Calgary, Calgary, AB, Canada; The Hospital for Sick Children, Toronto, ON, Canada; Queen’s University, Kingston, ON, Canada; Scarborough Health Network, Scarborough, ON, Canada; Boston Children’s Hospital, Boston, MA; Contrail Consulting Services Inc., Toronto, ON, Canada; Universite de Sherbrooke, Sherbrooke, QC, Canada; McGill University, Montreal, QC, Canada; Peadiatrics, The Hospital for Sick Children, Toronto, ON, Canada

## Abstract

**Background:**

Accurate optical diagnosis of colorectal polyps is essential for decision-making during colonoscopy, yet it remains a key skills gap among trainees and practicing endoscopists. To address this gap, we developed a cognitive simulation platform using high-fidelity polyp images to systematically develop endoscopists’ diagnostic and management decision-making skills through deliberate practice.

**Aims:**

To evaluate endoscopists’ learning curves for optical diagnosis and polyp management decision-making and to determine the number of cases required to achieve predefined performance benchmarks.

**Methods:**

Residents, fellows, and practicing endoscopists were recruited through training programs and national societies. Participants reviewed endoscopic images (in white light, narrow band imaging [NBI], and near focus), classified polyps using Paris, laterally spreading tumor [LST], NBI International Colorectal Endoscopic [NICE], and Japanese NBI Expert Team [JNET] classifications, predicted histology, and selected appropriate resection techniques. The primary outcome was learning rate, measured by a composite score of classification, histology prediction, and management decision-making. Learning curves were modeled using a deterministic signal processing model that allowed prediction of the number of cases needed to reach predefined accuracy benchmarks of 50%, 80%, and 90% for composite score, histology, and management.

**Results:**

A total of 248 participants completed 15,051 cases; 75 (30.2%) completed ≥50 cases and 46 (18.5%) completed all 135. Participants were attending physicians (n = 95, 38.3%), fellows (n = 133, 53.6%), residents (n = 6, 5.6%), and 2.4% unreported. Correct response rates and learning curves are shown in **Table 1** and **Figure 1**, respectively. The mean number (±SD) of cases to reach 50%, 80%, and 90% accuracy for composite score were 16.2(±12.2), 40.0(±7.9), and 81.9(±11.9), respectively. Similar mean numbers of cases were observed to achieve 50%, 80%, and 90% accuracy for histology (16.3±12.5, 37.3±7.2, and 71.2±11.3) and management (16.3±11.8, 37.3±6.9, and 71.3±9.7).

**Conclusions:**

This is the first report of a cognitive simulation-based training platform that effectively improves endoscopists’ colorectal polyp optical diagnosis and management skills. Rapid early learning with flattening of learning curves as participants approach high accuracy supports the validity of these findings.

**Funding Agencies:**

American Society for Gastrointestinal Endoscopy; Physician Services Incorporated